# Supplementary material for: Predictive feedback to V1 dynamically updates with sensory input
Source: Sci Rep. 2017 Nov 28;7:16538. doi: 10.1038/s41598-017-16093-y (PMC5705713; doi:10.1038/s41598-017-16093-y)
Supplement: Supplementary file 1 — Supplementary Information [file 41598_2017_16093_MOESM1_ESM.pdf]

Supplemental material: **Predictive feedback to V1 dynamically updates with sensory input**

Grace Edwards[1,2,3], Petra Vetter[1 & 4], Fiona McGruer[1], *Lucy S. Petro[1] & *Lars Muckli[1]

Affiliations

1. Centre for Cognitive Neuroimaging, Institute of Neuroscience and Psychology, College of Medical Veterinary and Life Sciences, University of Glasgow, 58 Hillhead Street, Glasgow, G12 8QB, UK.
2. Istituto Italiano di Tecnologia, Rovereto, Italy
3. Department of Psychology, Harvard University, Cambridge, USA
4. Department of Psychology, Royal Holloway, University of London, London, UK

These authors contributed equally to this work.
*Lucy S. Petro & Lars Muckli

Contributions
LM, GE and PV designed the research. GE, PV and FM performed the experiments. GE and FM preprocessed the data. GE analyzed the data. LM, GE and LP wrote the manuscript.

Competing financial interests
The authors declare no competing financial interests.

Corresponding author
Correspondence to Lars Muckli Lars.Muckli@glasgow.ac.uk

Acknowledgements
This work was supported by BBSRC grant (BB/G005044/1), ERC grant StG 2012_311751- ‘Brain reading of contextual feedback and predictions’ and Human Brain Project grant from the European Union’s Horizon 2020 Research and Innovation Programme under Grant Agreement No. 720270 (HBP SGA1) ‘Context-sensitive multisensory object recognition: a deep network model constrained by multi-level, multi-species data’.

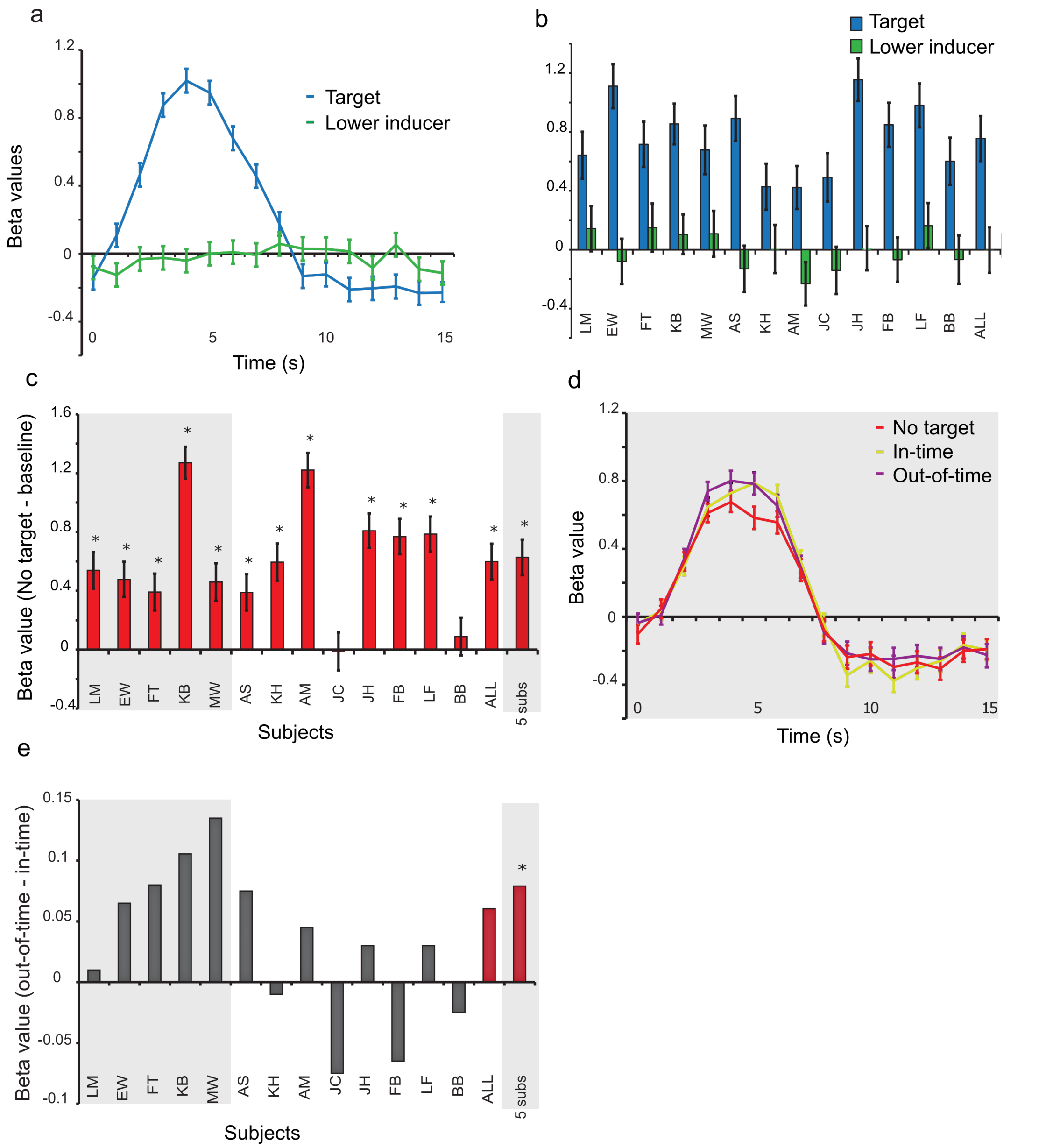

Supplemental Figure 1 – Related to Figure 3: Pilot Data. a) & b) Retinotopically defined target processing region in left V1 using contrast target mapping stimulus (blue) > lower inducer mapping stimulus (green). **a)** Group event-related BOLD responses to the target and lower inducer in target region of interest. **b)** Single subjects and group beta-values averaged over the peak BOLD activation of the target and the lower inducer in the target region of interest (*$p<0.05$). **c)** Difference in beta-values of 'apparent motion with no target' greater than 'baseline' conditions in target region. Beta – values averaged over peak activation (*$p<0.05$), Single subject and group beta-values shown, those with correctly positioned retinotopic activity highlighted by a grey shading. **d)** Group-averaged event-related BOLD responses to all apparent motion conditions in target processing region in left V1. Only the 5 subjects with correctly positioned retinotopic activity included in plot. **e)** Difference between in-time and out-of-time target trials at target processing region of left V1. Single subject, group-averaged (n=13, red), and group averaged data for subjects with valid retinotopic activity (n =5, red) is shown with grey shading.

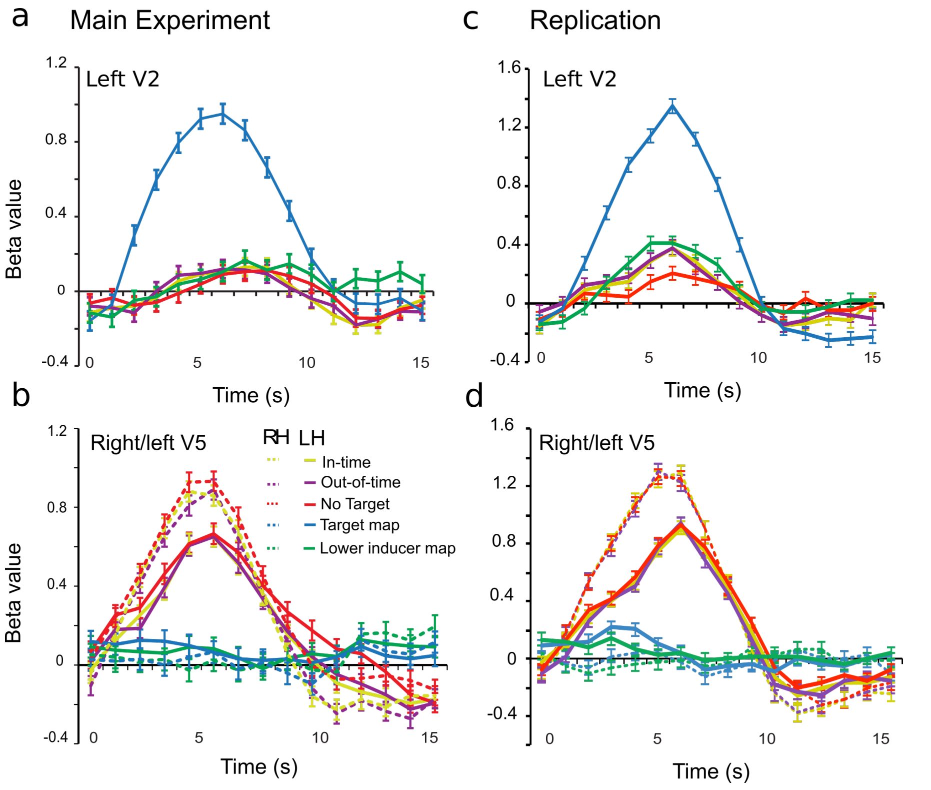

Supplemental Figure 2 – Related to Figure 3 & paragraphs: Apparent motion activity in left V2d & Predictive feedback from V5: BOLD Response to Apparent Motion Trials in the Main fMRI Experiment & Replication Apparent Motion Experiment – Left V2, Right V5 & Left V5. Main experiment data presented in top row, replication data in bottom row. **(a) & (c)** Group-averaged event related BOLD responses in left V2 region of interest (defined by contrast of target mapping stimulus > apparent motion mapping stimulus) **(b) & (d)** Group-averaged event related BOLD responses in right and left V5 regions of interest (regions defined by no-target apparent motion stimulus > baseline).

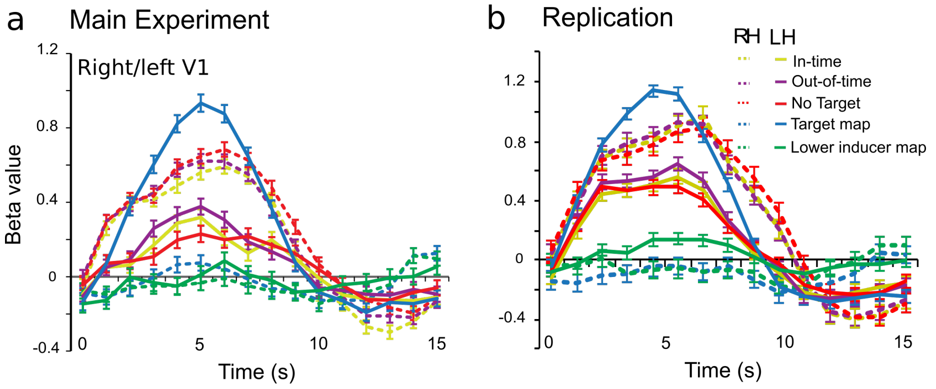

Supplemental Figure 3 – Related to Figure 3: BOLD Response to Apparent Motion Trials in Right V1 - Main & Replication fMRI experiments a) Group-averaged event-related BOLD responses in right V1 ROI defined by no target apparent motion > baseline contrast. Left V1 group averaged event-related BOLD response overlay for comparison of activity across trial. b) same as Supplemental figure 3a for replication experiment. Prior to the saccade, the motion illusion was processed in right V1; here we could not test for target-related effects because targets were only presented after the saccade. We did however expect to see apparent motion related illusory trace activity in pre-saccadic (right) V1. We found significant activation comprised of the apparent motion inducing stimuli and the illusory apparent motion percept in right V1 replicating previous findings (Main Experiment: in-time trials > baseline: $t(8)15.67, p<0.0001$, out-of-time trials > baseline: $t(8)16.18, p<0.0001$; Replication Experiment: in-time trials > baseline: $t(7)14.13$, $p<0.0001$, out-of-time trials > baseline: $t(7)13.50$, $p<0.0001$)).

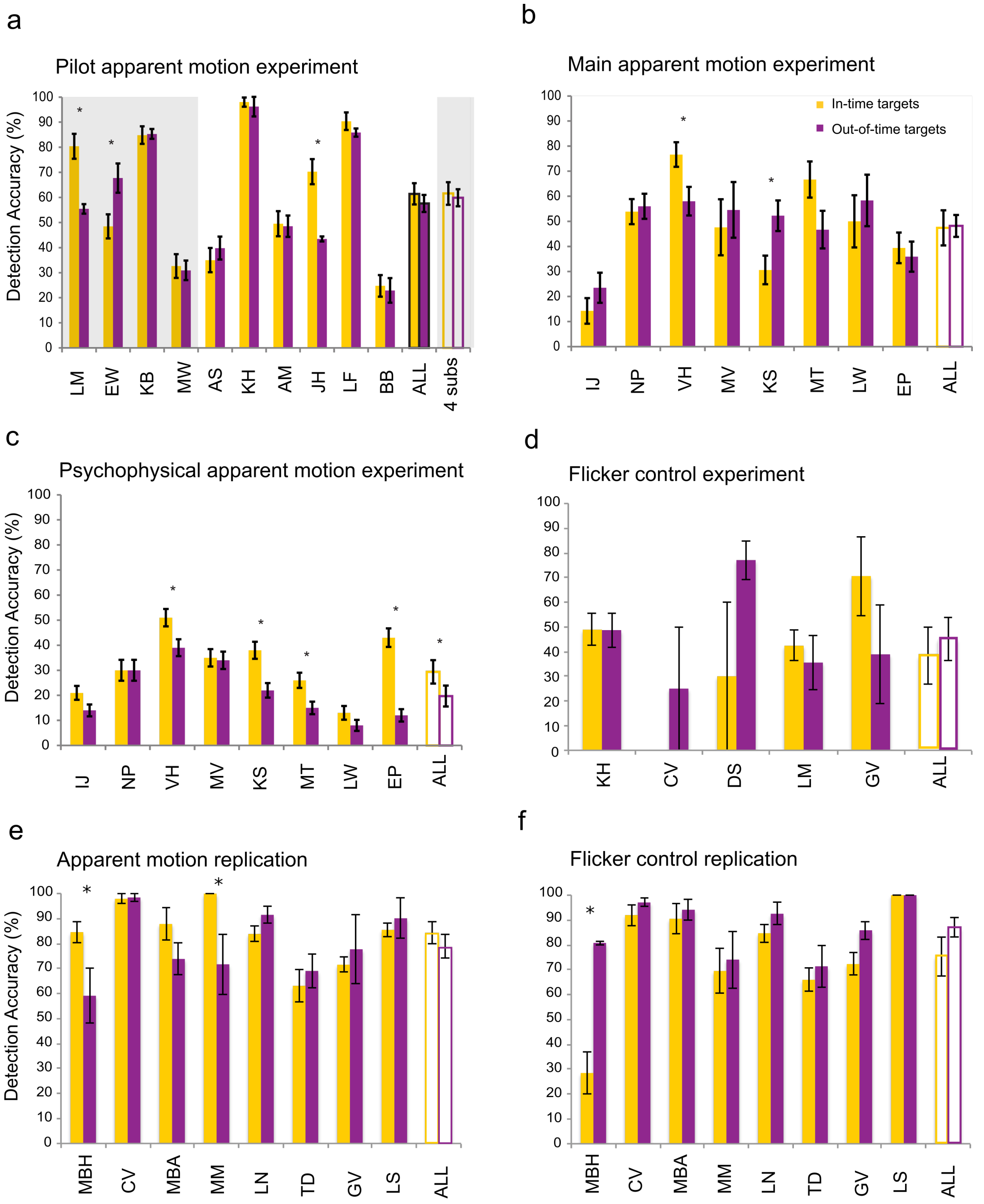

Supplemental Figure 4 - related to paragraph 'Extra-session psychophysical control experiment': Percentage detection accuracy for target – Single subject and group data. **4a-4f:** Yellow bars indicate in-time target detection accuracy and purple bars relate to out-of-time target detection accuracy. S**4a:** Behavioural data from pilot experiment. Grey bars indicate subjects without ventral activation, yellow/purple bars with black boundary are the whole group mean, and yellow/purple empty bars are the non-ventral activation subjects group only. Single subject (*p<0.02), average data (whole group (3 subjects removed due to technical issues with response recordings); n=10): t(9)0.898, p=0.392, group data for subjects with no ventral activation (grey background, n=4): t(3)0.192, p=0.860. **S4b:** Behavioural data from main fMRI experiment. Single subjects (*p<0.018), average data (n=8): t(7)-0.115, p=0.881. **S4c:** Extra-session psychophysical data. Single subjects (*p<0.02), average data t(7)3.073, p=0.015. **S4d:** Psychophysical data from flicker control: Single subjects (p>0.05), average data t(4)-0.492, p=0.648. **S4e:** Psychophysical data from apparent motion replication: Single

subjects (*p<0.03), average data t(7)1.011, p=0.346. **S4f:** Psychophysical data from flicker control replication: Single subjects (*p<0.0001), average data t(7)-1.932,p=0.095.

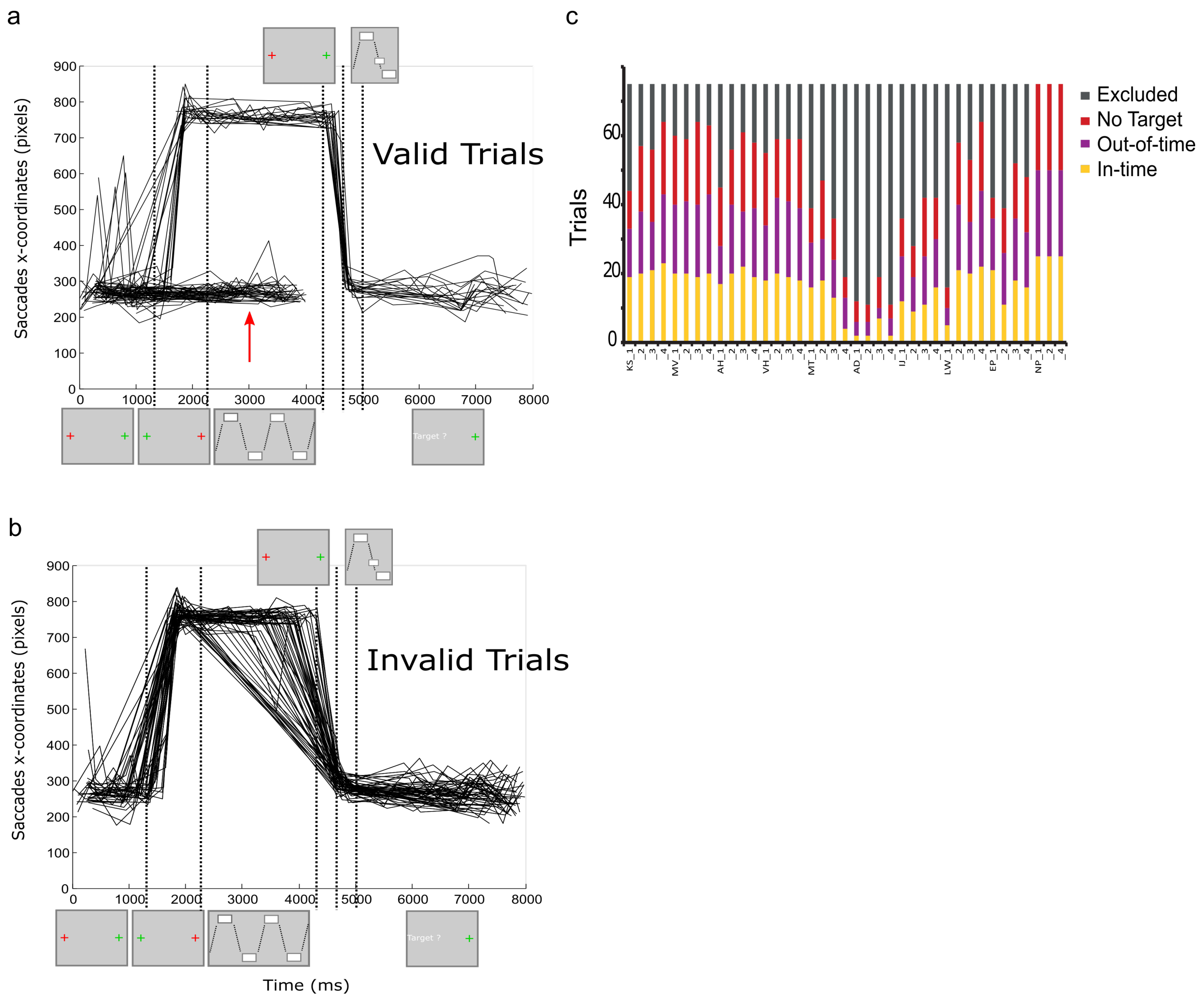

<u>Supplemental figure 5 – Related to methods: Saccade Criterion.</u> Saccade traces during each trial for one run (run 2) for one subject (IJ). Saccade criterion stipulates that the saccade lands 400ms after cue and that the saccade should travel further than 200 pixels at a leftward trajectory. **a)** trials that were included in the analysis (and the mapping trials where subjects remain fixated to the left for 4 seconds, red arrow). **b)** rejected trials where subjects made a saccade too early. **c)** proportion of trial rejection per subject per run.

<u>Supplemental Methods</u>

Pilot Study

Prior to our main fMRI experiment we piloted the study in 15 subjects using an identical paradigm. We presented apparent motion stimuli in the scanner using NordicNeuroLab goggles (screen resolution: 600 x 800). Visual degrees of stimuli were slightly different from the main experiment; the apparent motion inducers were 14.84° apart and were 3° visual angle in size. The fixation crosses were 10.3° from the center of the screen. The target stimulus was 2.3° visual angle in size. The apparent motion stimulus conditions, mapping stimuli, procedure, MRI data acquisition, and MRI analysis were performed the same as in the main fMRI experiment. We removed two subjects from the analysis due to excessive head-motion (leaving n= 13). Five of the remaining subjects showed expected retinotopically positioned activity in left V1. The other eight subjects showed signs of incorrect saccadic landing position due to retinotopically misplaced activity for the apparent motion conditions in left V1. We analysed the data of the five subjects with correctly positioned retinotopic activity (grey shading in Supplemental figure 1).

As in the main experiment, we tested for prediction-related activity on the illusory trace by first mapping a target region of interest (ROI) between the two apparent motion inducing stimuli. We presented a static target stimulus

in between the apparent motion inducers, and a static stimulus at the same location as the lower apparent motion inducer. To locate the target ROI, we used a GLM contrast (‘target’ region > ‘lower AM inducing stimulus’: beta-value = 0.947, t= 13.64, p<0.0001 Supplemental Figure 1a & 1b,). In this region, we compared the three apparent motion conditions for the 5 remaining subjects with valid eye movement data. We found evidence of activity along the illusory motion trace in left V1 post-saccade (no-target condition) in all 5 subjects (Supplemental Figure 1c; *p<0.05). This demonstrates that feedback related to the apparent motion illusion which built up in right V1 transferred to a new retinotopic region in left V1 with saccade. Providing further evidence that apparent motion predictions transferred to post-saccadic left V1, we found the out-of-time target condition amplified the activity along the apparent motion trace in comparison to the in-time target condition (in-time target trials: mean beta-value=0.67(SD=0.29); out-of-time target trials: mean beta-value=0.78(0.34); t(4)2.226, p=0.043, Supplemental Figure 1d & 1e). We interpret the amplification of BOLD signal for the out-of-time targets as a prediction error signal. This error signal occurs at the new retinotopic region in left V1 as the spatiotemporal prediction of apparent motion is relocated due to the saccadic eye-movement. The out-of-time target violates the transferred spatiotemporal apparent motion prediction resulting in the error signal. This evidence motivated the use of an Eyelink 1000 in the main fMRI experiment to enable the rejection of individual trials in which subjects performed incorrect eye movements. Although we observed the same pattern of results in the pilot and main experiments, we found smaller activity in all conditions in the main experiment. This decrease in beta values is caused by the increased distance of the subjects’ retina from the stimulus in the main experiment compared to the pilot. The stimulus was presented through goggles in the pilot whereas the stimulus was projected onto a screen in the MRI bore in the main experiment. Activity increases in the early visual cortex are found with a shorter viewing distance (e.g. [1–4]).

Supplemental References

1. Trotter, Y., Celebrini, S., Stricanne, B., Thorpe, S. & Imbert, M. Modulation of neural stereoscopic processing in primate area V1 by the viewing distance. *Science* **257,** 1279–1281 (1992).
2. Trotter, Y., Celebrini, S., Stricanne, B., Thorpe, S. & Imbert, M. Neural processing of stereopsis as a function of viewing distance in primate visual cortical area V1. *J. Neurophysiol.* **76,** 2872–2885 (1996).
3. Jaschinski-Kruza, W. Eyestrain in VDU Users: Viewing Distance and the Resting Position of Ocular Muscles. *Hum. Factors J. Hum. Factors Ergon. Soc.* **33,** 69–83 (1991).
4. Sperandio, I., Chouinard, P. A. & Goodale, M. A. Retinotopic activity in V1 reflects the perceived and not the retinal size of an afterimage. *Nat. Neurosci.* **15,** 540–542 (2012).
